# Supplementary material for: Trehalose metabolism confers developmental robustness and stability in Drosophila by regulating glucose homeostasis
Source: Commun Biol. 2020 Apr 7;3:170. doi: 10.1038/s42003-020-0889-1 (PMC7138798; doi:10.1038/s42003-020-0889-1)
Supplement: Supplementary file 2 — Description of Additional Supplementary Files [file 42003_2020_889_MOESM2_ESM.pdf]

Description of Additional Supplementary Files

File Name: **Supplementary Data 1**
Description: The file contains the raw data underlying each graph and statistical results
